# The Vitamin B_1_ and B_12_ Required by the Marine Dinoflagellate *Lingulodinium polyedrum* Can be Provided by its Associated Bacterial Community in Culture

**DOI:** 10.3389/fmicb.2016.00560

**Published:** 2016-05-06

**Authors:** Ricardo Cruz-López, Helmut Maske

**Affiliations:** Oceanografía Biológica, Centro de Investigación Científica y de Educación Superior de EnsenadaEnsenada, Mexico

**Keywords:** B vitamin auxotrophy, dinoflagellate–bacteria interactions, fluorescence *in situ* hybridization (FISH), 16S rDNA, Illumina-MiSeq sequencing

## Abstract

In this study we established the B_1_ and B_12_ vitamin requirement of the dinoflagellate *Lingulodinium polyedrum* and the vitamin supply by its associated bacterial community. In previous field studies the B_1_ and B_12_ demand of this species was suggested but not experimentally verified. When the axenic vitamin un-supplemented culture (B-ns) of *L. polyedrum* was inoculated with a coastal bacterial community, the dinoflagellate’s vitamin growth limitation was overcome, reaching the same growth rates as the culture growing in vitamin B_1_B_7_B_12_-supplemented (B-s) medium. Measured B_12_ concentrations in the B-s and B-ns cultures were both higher than typical coastal concentrations and B_12_ in the B-s culture was higher than in the B-ns culture. In both B-s and B-ns cultures, the probability of dinoflagellate cells having bacteria attached to the cell surface was similar and in both cultures an average of six bacteria were attached to each dinoflagellate cell. In the B-ns culture the free bacterial community showed significantly higher cell abundance suggesting that unattached bacteria supplied the vitamins. The fluorescence *in situ* hybridization (FISH) protocol allowed the quantification and identification of three bacterial groups in the same samples of the free and attached epibiotic bacteria for both treatments. The relative composition of these groups was not significantly different and was dominated by Alphaproteobacteria (>89%). To complement the FISH counts, 16S rDNA sequencing targeting the V3–V4 regions was performed using Illumina-MiSeq technology. For both vitamin amendments, the dominant group found was Alphaproteobacteria similar to FISH, but the percentage of Alphaproteobacteria varied between 50 and 95%. Alphaproteobacteria were mainly represented by *Marivita* sp., a member of the *Roseobacter* clade, followed by the Gammaproteobacterium *Marinobacter flavimaris*. Our results show that *L. polyedrum* is a B_1_ and B_12_ auxotroph, and acquire both vitamins from the associated bacterial community in sufficient quantity to sustain the maximum growth rate.

## Introduction

Culture-based (Tang et al., 2010) and field studies (Bertrand et al., 2007; Gobler et al., 2007; Koch et al., 2011, 2012) have supported long standing hypothesis that vitamin availability can have an impact on phytoplankton growth and community composition (Droop, 2007). A majority of eukaryotic phytoplankton requires exogenous B vitamins, hence being B vitamin auxotroph. They are lacking the biosynthetic pathways to produce them or alternative pathways to bypass the need for the vitamin as in the case of B_12_. Of the examined species 54% required vitamin B_12_ (cobalamin; hereafter B_12_), 27% required vitamin B_1_ (thiamine; hereafter B_1_) and 8% required vitamin B_7_ (biotin; hereafter B_7_) (Tang et al., 2010). B_12_ is essential for the synthesis of amino acids, deoxyriboses, and the reduction and transfer of single carbon fragments in many biochemical pathways. B_1_ plays a pivotal role in intermediary carbon metabolism and is a cofactor for a number of enzymes involved in primary carbohydrate and branched-chain amino acid metabolism. B_7_ is a cofactor for several essential carboxylase enzymes, including acetyl coenzyme A (CoA) carboxylase, which is involved in fatty acid synthesis, and so is universally required (Croft et al., 2006; Tang et al., 2010).

Dinoflagellates are among the most abundant eukaryotic phytoplankton in freshwater and coastal systems (Moustafa et al., 2010). Of the 45 examined dinoflagellate species, those species involved in harmful algal bloom events, 100% required B_12_, 78% B_1_ and 32% B_7_ (Tang et al., 2010). Available genomic data indicate that some heterotrophic bacteria and archaea, as well marine cyanobacteria are vitamin producers (Bonnet et al., 2010; Sañudo-Wilhelmy et al., 2014). Many dinoflagellates are mixotrophs (Burkholder et al., 2008), therefore they could acquire their B vitamins from the environment either through phagotrophy (Jeong et al., 2005), active uptake from the soluble fraction (Bertrand et al., 2007; Kazamia et al., 2012) or through episymbiosis (Croft et al., 2005; Wagner-Döbler et al., 2010; Kazamia et al., 2012; Kuo and Lin, 2013; Xie et al., 2013). The relative contribution of these different mechanisms to vitamin acquisition of dinoflagellates is not known but could help in the understanding of dinoflagellate ecology and the possible role of vitamins in bloom development.

*Lingulodinium polyedrum* is a dinoflagellate with a mixotrophic lifestyle (Jeong et al., 2005) that is recurrently forming blooms along the coast of southern California and northern Baja California (Peña-Manjarrez et al., 2005). Although its physiology (Hastings, 2007; Beauchemin et al., 2012) and microbial ecology (Mayali et al., 2008, 2010, 2011) have been previously studied, its vitamin auxotrophy has only been inferred from oceanographic observations (Carlucci, 1970), but has not been experimentally established. Here we investigate the role of vitamins and bacteria in the autecology of *L. polyedrum*, using axenic and non-axenic cultures of *L. polyedrum* under different combinations of multiple and single vitamin limitation.

To document the association of a natural bacterial community in *L. polyedrum* cultures under B_1_B_7_B_12_-supplemented (hereafter B-s) and B_1_B_7_B_12_-not supplemented (hereafter B-ns) cultures, we employed a FISH and digital imaging approach to quantify free and attached bacteria, and 16S rDNA sequencing targeting V3–V4 regions looking for phylogenetic composition. To complement the information on the B_12_ synthesis, we quantified the B_12_ in both vitamin amendments.

## Materials and Methods

### Strain and Growth Conditions

Coastal seawater was collected off Ensenada (31.671° N, 116.693° W; Ensenada, México) treated with activated charcoal, filtered through GF/F, and 0.22-mm pore-size cartridge filter (Pall corporation) and stored in the dark at room temperature to age for at least 2 months. Aged seawater was sparged with CO_2_ (5 min per 1 L of seawater), autoclaved for 15 min and then equilibrated with air. Non-axenic *L. polyedrum* HJ culture (Latz Laboratory, UCSD-SIO) was grown in L1 medium (National Center for Marine Algae and Microbiota, Boothbay, ME, USA) prepared with aged oceanic water under 12:12 h light/dark cycle at an irradiance level of 100 μmol m^2^ s^–1^ and a temperature of 20°C. To make the culture axenic, *L. polyedrum* cultures were incubated with 1 ml of antibiotic solution (Penicillin, 5,000 U; Streptomycin, 5 mg ml^–1^; Neomycin, 10 mg ml^–1^. Sigma–Aldrich, P4083-100ML) for 50 ml of culture during 24 h, rinsed with L1 medium, and repeated three times each step. Bacterial presence in the *L. polyedrum* culture was checked by staining with the nucleic acid-specific stain 4′,6-diamino-2-phenylindole (DAPI,1 μg ml^–1^) and quantification using epifluorescence microscopy (Axioskope II plus, Carl Zeiss, Oberkochen, Germany) connected by liquid light guide to a 175 W xenon arc lamp (Lambda LS, Sutter), with optical filtering (Excitation, 360 nm/Dichroic, 395 nm / Emission, >397 nm; Semrock and Zeiss) under X100 objective lens (Plan-Apochromat, Carl Zeiss). The axenic status was declared when after three antibiotic rounds and sterile medium washes we could not detect bacteria in the culture through epifluorescence microscopy. Semi-continuous cultures were transferred approximately weekly using 10 × dilutions.

### Qualitative Assessment of B_1_, B_7_, and B_12_ Dinoflagellate Auxotrophy

Bacteria are a potential source of B_1_, B_7_, and B_12_, making it necessary to establish axenic cultures before testing vitamin auxotrophy. To test the vitamin auxotrophic status of *L. polyedrum*, cultures (*n* = 3) were grown semi-continuously in 15 ml glass test tubes with silicon stoppers. Cultures were acclimated by five semi-continuous transfers during 5 weeks. The medium for *L. polyedrum* axenic cultures was supplemented either with the L1 vitamin mix (B_1_, 296000; B_7_, 2050; B_12_, 370; pmol L^–1^), or with the following combinations of vitamins, B_1_+B_12_, B_1_+B_7_, and B_7_+B_12_ at the same concentration (B_1_, Sigma–Aldrich; B_7_, Sigma–Aldrich; B_12_, Sigma–Aldrich). Cell growth rate was monitored by mixing first the cultures with an inclined rotating test tube holder (10 rpm) before measuring *in vivo* chlorophyll *a* fluorescence using a Turner Designs 10-000 fluorometer at the midpoint of the light phase. The specific growth rate (μ) was estimated according to the equation μ = ln(N2/N1)/(t2–t1): where N1 and N2 was *in vivo* chlorophyll *a* fluorescence in relative units (r. u.) at time 1 (t1) and time 2 (t2) respectively. Vitamin auxotrophy was declared when a culture ceased to grow in the absence of vitamins while growth persisted in parallel control treatments with added vitamin.

### Qualitative Assessment of B_1_ and B_12_ Synthesis from the Bacterial Community

The axenic *L. polyedrum* culture (*n* = 3) was inoculated with a prokaryotic community obtained by filtering (0.8 μm polycarbonate filter) rocky intertidal seawater (31.861755 °N, 116.668097°W; Ensenada, México). *L. polyedrum* culture lines were divided into B-s and B-ns cultures, and acclimated by culture transfer for 6 months to ensure depletion of the initial vitamin present and to be sure that the persisting microbial community had the potential to synthesize vitamins. The specific growth rate was monitored as described above, taking the day eight as t2 and day zero as t1.

### Cell Fixation, Immobilization, and Embedding

Five mililiter of *L. polyedrum* cells from B-s and B-ns cultures were harvested at lag, exponential and stationary phase and fixed with paraformaldehyde-PBS at a final concentration of 1% for 12 h at 4°C. For attached bacteria, fixed cells were immobilized onto an 8.0 μm pore size, 25 μm-diameter Nucleopore filter (Whatman International, Ltd., Maidstone, England) using a pressure difference of <3.3 kPa to avoid cell disintegration, and rinsed with phosphate-buffered saline (PBS,0.1 M NaCl, 2 mM KCl, 4 mM Na_2_HPO_4_, pH 8.1; Palacios and Marín, 2008). For free-living bacteria, the fraction which passed through 8.0 μm pore size filter was collected on 0.2 μm pore size, 25 mm-diameter Nucleopore filter (Whatman International, Ltd., Maidstone, England) and rinsed with PBS. The cells collected on the 8.0 μm filter were covered with 13 μl of low-melting point agarose (0.05%, LMA) (BioRad, 161-3111) at 55°C, dried for 15 min at 37°C, then LMA was added again and the filter dried as previously.

### Fluorescence *In Situ* Hybridization

All *in situ* hybridizations were performed as described in Cruz-López and Maske (2014). In brief, before hybridization, bacterial cells were partially digested with 400,000 U ml^–1^ lysozyme (Sigma, L6876) dissolved in buffer containing 100 mM Tris-HCl, 50 mM EDTA, pH 8.0 for 1 h at 37°C. The enzyme reaction was stopped by rinsing the filter three times with 5 ml sterile water for 1 min at 4°C. Fixed and embedded samples were hybridized with a buffer containing 900 mM NaCl, 20 mM Tris-HCl, 0.02% SDS, pH 8.0. When probes with different stringency optima were applied to the same sample, sequential hybridization was performed, beginning with the probes requiring the most stringent conditions. Probes and hybridization conditions are listed in **Table 1**. Hybridizations containing 1 μl of probe for every 20 μl of buffer (final probe concentration = 25 ng μl^–1^) were performed at 46°C for 2 h. After this, filters were washed with pre-warmed (48°C) buffer (900 mM NaCl, 20 mM Tris-HCl, 0.02% SDS, 5 μM EDTA) for 15 min and rinsed for 5 min in distilled H_2_O. To be able to localize the theca and bacterial cells, we used Calcofluor (Sigma-Aldrich, México City, México) (1 μg ml^–1^) and for DNA staining 4,6-diamino-2-phenylindole (DAPI Molecular Probes) (1 μl ml^–1^), incubating 5 min in the dark at room temperature. Filters were rinsed twice with sterile H_2_O for 5 min, dried and mounted in antifade reagent (Patel et al., 2007) and covered with cover slip.

**Table 1:** Oligonucleotide probes used in this study.

Probe	Target group	Sequence (5′-3′)	Target site^a^	% Formamide in ISH^b^ buffer	Reference
EUB338	Bacteria	GCTGCCTCCCGTAGGAGT	16S (338–355)^c^	0–50	Amann et al., 1990
NON338	Negative control	ACTCCTACGGGAGGCAGC	16S (338–355)^c^		Amann et al., 1990
ALF968	Alphaproteobacteria	GGTAAGGTTCTGCGCGTT	16S (968–986)^e^	20	Glöckner et al., 1999
CF319a	Bacteroidetes	TGGTCCGTGTCTCAGTAC	16S (319–336)^d^	35	Manz et al., 1996
GAM42a	Gammaproteobacteria	GCCTTCCCACATCGTTT	23S (1027–1043)^c^	35	Glöckner et al., 1999

^a^*E. coli* numbering; ^b^*In situ* hybridization; ^c^Cy3; ^d^ATTO425; ^e^Alexa594.

### Visualization

The epifluorescence microscope (Axioskope II plus, Carl Zeiss, Oberkochen, Germany; oil immersion X100 objective, Plan-Apochromat, Carl Zeiss; 175 W xenon arc lamp; Lambda LS, Sutter connected through a liquid light guide) was used with a triple Sedat filter, a dichroic filter with three transmission bands. Excitation and emission spectra were controlled by filter wheels (Lambda 10-3, Sutter). Images were captured with a cooled CCD camera (Clara E, Andor) with 10 ms integration time. Optical stacks, 2.0 μm focal distance between images, were acquired with a computer controlled focusing stage (Focus Drive, Ludl Electronic Products, Hawthorne, NY, USA) and Micro-Manager (version 1.3.40, Vale Lab, UCSF) that controlled filter selection and the focusing stage. The images were processed in ImageJ (Schneider et al., 2012). For each spectral channel a summary image was composed by selecting the pixels of maximum intensity within the stack, and using the ‘Find Edges’ and ‘Despeckle’ functions to reduce images noise (Cruz-López and Maske, 2014).

### 16S rRNA Gene Amplicon Sequencing

The free and attached bacterial communities from B-s and B-ns cultures were characterized and compared using barcoded high-throughput amplicon sequencing of the bacterial 16S rDNA. Four samples with no replicates were sequenced: (1) 50 ml of *L. polyedrum* cells from B-s and B-ns cultures were harvested at mid-exponential phase, and pre-filtered with (a) 8.0 μm pore size, 47 mm-diameter Nucleopore filter (Whatman International, Ltd., Maidstone, England) using a pressure difference of <3.3 kPa to avoid cell disintegration, with a second filtration step with (b) 0.4 μm pore size, 47 mm-diameter polycarbonate filter (Whatman International, Ltd., Maidstone, England) to recover the free-bacterial fraction, and (2) The first filter (a) containing the dinoflagellate cells were treated with 10 mM *N*-acetyl cysteine (NAC; Sigma) (PBS – 0.2 μM calcium chloride, 0.5 mM magnesium chloride, 15 mM glucose) for 1 h with agitation (70 rpm) at room temperature to detach adhered bacteria (Barr et al., 2013). The detached bacterial cells were collected onto a 0.4 μm pore size, 47 mm-diameter polycarbonate filter. Filters containing both free and detached bacterial communities were processed by the Research and Testing Laboratory (RTL, Lubbock, TX, USA), including DNA extraction. The bacterial hypervariable regions V3-V4 of the 16S rRNA gene were PCR amplified using the forward 341F (CCTACGGGNGGCWGCAG) and the reverse 805R (GACTACHVGGGTATCTAATCC) primers set (Herlemann et al., 2011) followed by sequencing on the MiSeq platform (Illumina Inc., USA). MiSeq reads were quality checked and paired reads joined using PEAR (Zhang et al., 2013), dereplicated using USEARCH (Edgar, 2010), OTU selected using UPARSE (Edgar, 2013) and chimera checked using UCHIME (Edgar et al., 2011) executed in *de novo* mode. Generated sequences then were run against the RDP classifier employing the NCBI database. All downstream analysis were done in the RTL facility using their standard pipeline. The MiSeq reads have been deposited in the NCBI Sequence Read Archive with accession number SRP071004.

### Dissolved B_12_ Quantification

B_12_ was quantified during the mid-exponential phase in non-axenic B-s and B-ns cultures. Twenty mililiter of *L. polyedrum* cultures were harvested, and pre-filtered with 8.0 μm pore size, followed by a filtration step with 0.45 μm pore size, both 47 mm-diameter Nucleopore filter (Whatman International, Ltd., Maidstone, England) using a pressure difference of <3.3 kPa to avoid cell disintegration. B_12_ was pre-concentrated by solid phase (RP-C18) extraction according to Okbamichael and Sañudo-Wilhelmy (2004), the column was eluted with 5 ml methanol, the methanol was evaporated at 60°C with vacuum, and 50 μl of the concentrate was injected into the ELISA well plate for quantification (Immunolab GmbH, B12-E01. Kassel, Germany) according to Zhu et al. (2011).

### Statistical Analysis

One-way ANOVA was used to compare growth rate curves of *L. polyedrum* under B-s and B-ns cultures, and to compare the B_12_ synthesis from both bacterial communities. Since the distribution of the number of attached bacteria per dinoflagellate cell was not normal, a Kruskal–Wallis test was used to assess the significance (α = 0.05) in the number of attached bacterial during the *L. polyedrum* growth curve. All analysis were performed using the STATISTICA 7.1v software (Stat Soft Inc., USA).

## Results

### *Lingulodinium polyedrum* Auxotrophy for B Vitamins

**FIGURE 1:**
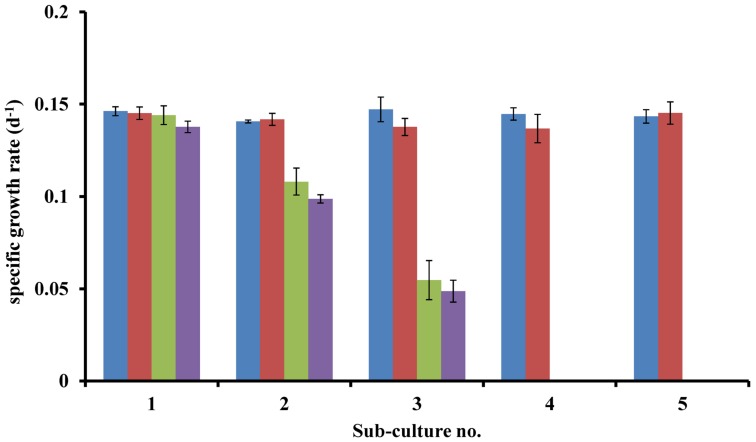
**Specific growth rates of axenic ***L. polyedrum*** grown in: (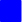) B_1_B_7_B_12-s_; (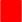) B_1_B_12-s_; (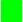) B_1_B_7-s_; (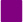) B_7_B_12-s_ cultures**.

*Lingulodinium polyedrum* was grown axenically with (B-s) and without (B-ns) adding the L1 vitamin mix. B-s cultures continued growing after five subcultures, B-ns cultures started to show slower growth rates after one transfer, but only after the third transfer, the B-ns cultures stopped growing. To establish which B vitamins were limiting the growth of *L. polyedrum* we prepared cultures containing (B_1_B_7_B_12_), (B_1_B_12_), (B_1_B_7_) and (B_7_B_12_). Under B_1_B_7_B_12_-s condition, cultures continued growing after five subcultures. In the B_1_B_12_-s condition cultures showed the same growth rate as the B_1_B_7_B_12_-s condition, whereas in B_1_B_7_-s and B_7_B_12_-s condition, the cultures ceased to grow. These experiments were repeated three times with the same result (**Figure 1**).

### Co-Culture of *L. polyedrum* with a Natural Bacterial Community

**FIGURE 2:**
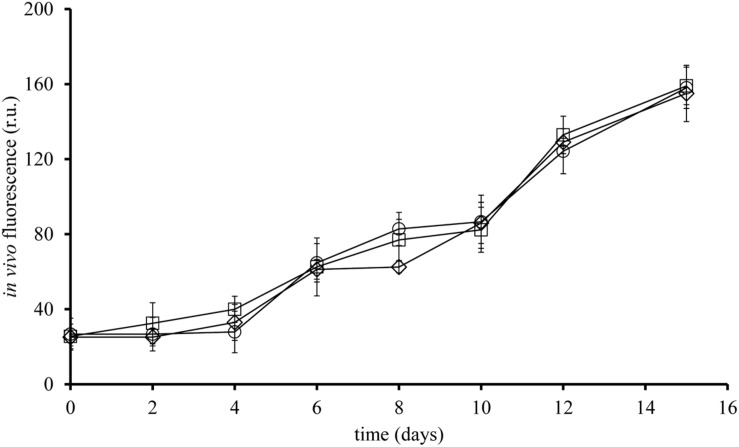
**Growth curve of ***L. polyedrum***, axenic or in co-culture with a natural marine bacterial community.** (□) axenic, B-s; (○) non-axenic, B-ns; (♢) non-axenic, B-ns. Each curve is the results of three culture replicates with the standard deviation indicated by vertical bars.

**FIGURE 3:**
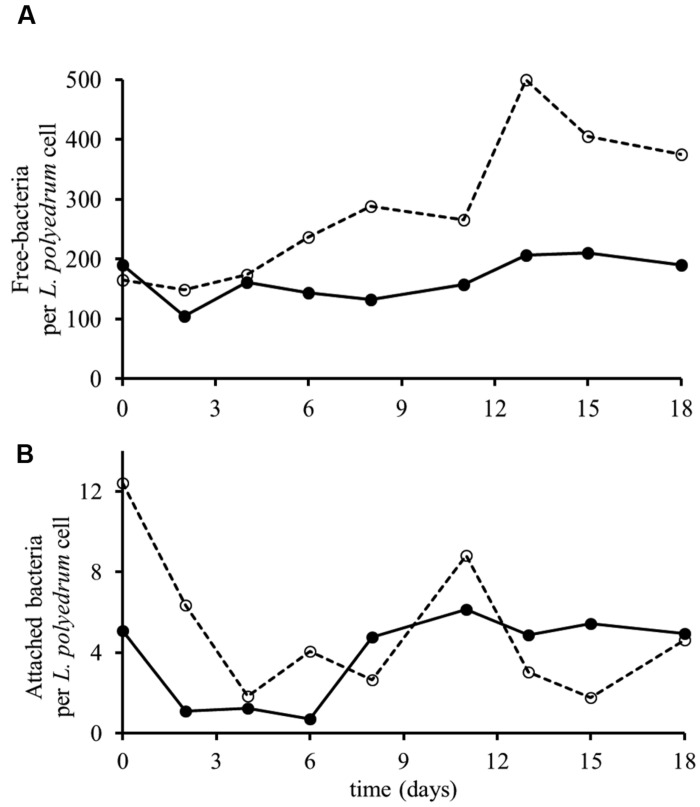
**Growth of ***L. polyedrum*** and associated bacteria culture under B-s (–•–) and B-ns (–○–) conditions. (A)** Ratio of freely suspended bacteria to *L. polyedrum* cells. **(B)** Average number of bacteria attached to those *L. polyedrum* cells that had at least one bacteria attached. *n* = 50 dinoflagellate cells.

Non-axenic *L. polyedrum* culture in B-ns condition could maintain growth through more than five culture transfers at growth rates similar (*p* > 0.05) to the axenic and the non-axenic, B-s cultures (**Figure 2**). These results suggest that the bacteria in the non-axenic culture could provide sufficient amounts of vitamins to sustain *L. polyedrum* growth. In the non-axenic cultures the concentration of freely suspended bacteria in the B-ns culture was significantly higher than in the B-s culture (*p <* 0.05; **Figure 3A**, **Supplementary Figure S1**). The mean number of attached bacteria ranged from 1 to 6 in B-s culture and from 1 to 12 in B-ns culture; in both types of non-axenic cultures the probability of *L. polyedrum* cells to have bacteria attached or the average number of bacteria attached to *L. polyedrum* cells were not significantly different (*p* > 0.05; **Figure 3B**).

### Free and Attached Bacterial Community by FISH

**FIGURE 4:**
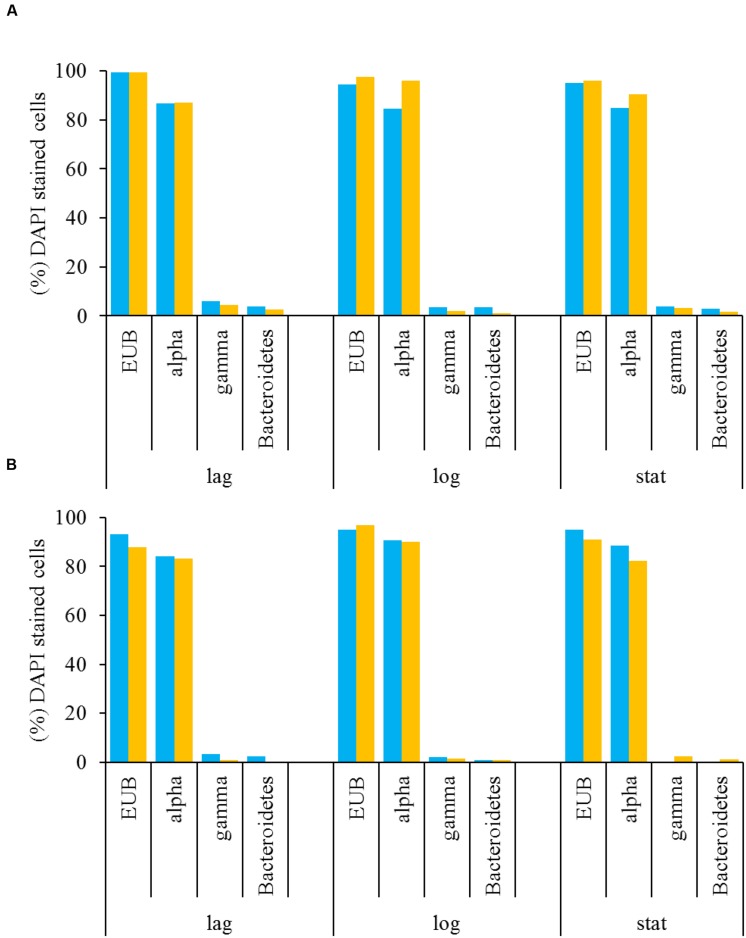
**Free-living (A) and attached (B) bacteria associated with ***L. polyedrum*** in B-ns (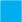) and B-s (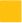) cultures during different culture phases (lag, mid-exponential and stationary phases) given in percentages of the total number of DAPI-stained cells.** Specific bacterial groups were quantified by FISH hybridized with the four probes listed in **Table 1**.

A multiprobe FISH protocol was used to identify the major bacterial groups in *L. polyedrum* cultures that were either freely suspended or attached to the host cells (Cruz-López and Maske, 2014). These results showed that Alphaproteobacteria were abundant in the free and attached fractions of B-s and B-ns cultures. In both vitamin treatments Alphaproteobacteria comprised on average 80% of the bacterial community, while Gammaproteobacteria and Bacteroidetes were scarcely detected (**Figures 4A,B**). As stated in Biegala et al. (2002) when working with phytoplankton it is critical to use group-specific probes to discriminate the false positives coming from the plastids. In our samples we could discriminate the eubacterial probe (EUB338) from the group-specific probes this allowed us to confirm the group specific probes with bacteria attached to *L. polyedrum* cells.

### Free and Attached Bacterial Community by 16S Amplicons

We assessed the relative abundance of bacterial taxa at the level of phylum, class, genus and species for each sample. Four samples with no replicates were sequenced: the attached and the free living bacterial community of B-s and B-ns cultures.

**FIGURE 5:**
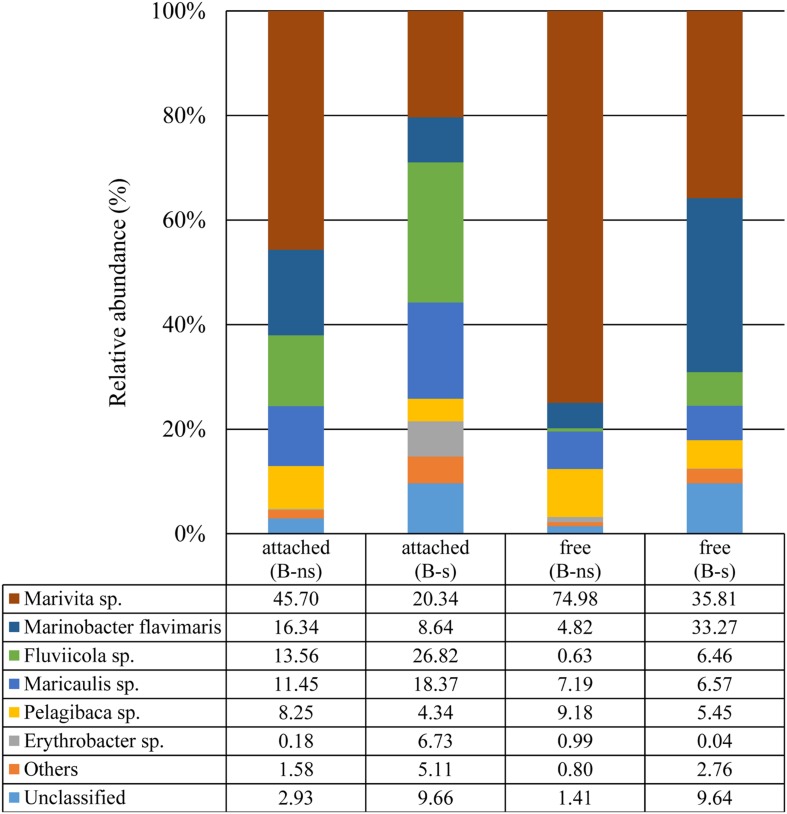
**Taxonomic classification of bacterial 16S rDNA (V3–V4) Illumina reads at species level**.

In the B-s culture, most of the free-bacterial community reads were assigned to the class Alphaproteobacteria (49.6%), with *Marivita* sp. as the dominant species (35.8%), followed by *Maricaulis* sp. (6.5%) and *Pelagibaca* sp. (5.4%). The class Gammaproteobacteria represented 35.8%, with *Marinobacter flavimaris* as the dominant species (33.2%). Other phyla presented were Actinobacteria, Planctomycetes, Cyanobacteria and Firmicutes representing the 2.76%, while the unclassified reads represented the 9.64% (**Figure 5**).

In the B-ns culture, most of the free-bacterial community reads were assigned to the class Alphaproteobacteria comprising 93.6% of the detected sequences, with *Marivita* sp. as the dominant species in the sample (74.9%) followed by *Pelagibaca* sp. (9.1%), *Maricaulis* sp. (7.1%). The class Gammaproteobacteria represented 5.1%, dominated by *Marinobacter flavimaris* (4.8%). Other phyla presented were Actinobacteria, Planctomycetes, Cyanobacteria and Firmicutes representing less than 1%, while the unclassified reads represented the 1.4% (**Figure 5**)

In the B-s culture, even though the attached bacterial community was dominated by the Alphaproteobacteria (51.2%), the dominant species was the Bacteroidetes *Flaviicola* sp. (26.8%), followed by the Alphaproteobacteria *Marivita* sp. (20.3%), *Maricaulis* sp. (18.3%), *Erythrobacter* sp. (6.7%), *Pelagibaca* sp. (4.3%), and the Gammaproteobacteria *Marinobacter flavimaris* (8.6%). Other phyla presented were Planctomycetes, Actinobacteria, Firmicutes and Cyanobacteria representing 5.11% of the reads, while the unclassified reads represented 9.6% (**Figure 5**).

In the B-ns culture, the attached community was dominated by the class Alphaproteobacteria (66.3%), the dominant observed species were the Alphaproteobacteria *Marivita* sp. (45.7%), the Gammaproteobacteria *Marinobacter flavimaris* (16.3%), and the Bacteroidetes *Flaviicola* sp. (13.5%) (**Figure 5**) followed by the alphaproteobacterial species *Maricaulis* sp. (11.4%) and *Pelagibaca* sp. (8.2%) (**Figure 5**). Other phyla presented were Planctomycetes, Actinobacteria, Firmicutes and Cyanobacteria representing 1.58% of the reads, while the unclassified reads represented 2.9% (**Figure 5**).

### Dissolved B_12_ Quantification

After 5 days during the mid-exponential phase, we quantified B_12_ in B-s and B-ns cultures; our results show that the synthesis of B_12_ from the microbial community varies between conditions. The initial B_12_ concentration in the L1 medium is 370 pmol L^–1^ (Guillard, 1975). In our cultures, we quantified the B_12_ during the exponential phase, in the B-s culture, the B_12_ was 26.3 ± 2.8 pmol L^–1^ (*n* = 3), and in the B-ns culture was 14.4 ± 7.6 pmol L^–1^ (*n* = 3).

In both cases, the differences of B_12_ in the cultures (*p* < 0.05) are the balance between the consumption of the *L. polyedrum* population and the synthesis and excretion from the microbial community. The vitamin mix of the L1 medium includes B_1_ and B_7_, and below we only discuss B_12_ because we could quantify its concentration.

## Discussion

### *Lingulodinium polyedrum* and Vitamin Auxotrophy

Phytoplankton vitamin B auxotrophy has been previously observed in cultures (reviewed by Droop, 2007) and in natural phytoplankton assemblages in coastal areas (Sañudo-Wilhelmy et al., 2006: Gobler et al., 2007; Koch et al., 2012). Here we address vitamin auxotrophy of *L. polyedrum* and its vitamin supply by natural bacterial communities. *L. polyedrum* was chosen because it forms coastal red tides and because previous studies had demonstrated high bacterial abundance and diversity of attached bacteria (Fandino et al., 2001; Mayali et al., 2011). Carlucci (1970) interpreted phytoplankton and B_12_ data from coastal waters of S. California and concluded that *L. polyedrum* was B_12_ auxotroph, but *L. polyedrum* vitamin B auxotrophy has not been previously tested experimentally. In our axenic cultures the sequential transfer into vitamin B free media led to a continuous reduction in growth rates until growth stopped. The continued growth of the first subcultures was probably supported by the vitamin present in the inoculum used for culture transfer, either in the medium or intracellularly. Further culture experiments demonstrated B_1_ and B_12_ auxotrophy of *L. polyedrum* (**Figure 1**) similar to what has been reported for other dinoflagellate species (Tang et al., 2010). B_1_ and B_12_ behaved like independent limiting factors, both vitamins were necessary to support growth.

*Lingulodinium polyedrum* is expected to be mixotrophic, potentially allowing for different modes of vitamin uptake; through osmotrophy, phagotrophy (Jeong et al., 2005) or through the episymbiosis with heterotrophic bacteria attached to cells (Croft et al., 2005). In our cultures the probability of bacterial attachment to *L. polyedrum* cells and the number of attached bacteria was not significantly different in B-ns and B-s cultures of *L. polyedrum* which argues against episymbiosis. The ratio of freely suspended bacteria to *L. polyedrum* cells did significantly increase in B-ns cultures, suggesting that vitamins for *L. polyedrum* growth were provided by part of the free bacterial community and then the dissolved vitamin was taken up by *L. polyedrum* and the auxotrophic part of the bacterial community.

The measured concentration of B_12_ in the B-ns non axenic culture was close to 50% lower than the B-s culture, but despite that, both concentrations are in the range of highly productive coastal systems (reviewed in Sañudo-Wilhelmy et al., 2014), Because culture concentrations were similar its seems probable that the B_12_ concentrations did not limit the *L. polyedrum* growth rate in culture. The concentrations in the culture without vitamin added were measured during exponential phase and represent the equilibrium concentration between the continuous supply from the bacterial consortium and the uptake by the dinoflagellate. In this case the bacterial consortium includes B_12_ producers and consumers with an overall B_12_ overproduction. It should be considered that the B-s culture received not only B_12_ but in addition B_1_ and B_7_. Because *L. polyedrum* is B_1_ auxotroph the addition of this vitamin might influence the outcome of the equilibrium B_12_ concentration.

Although we found no increase in bacteria attached to *L. polyedrum*, the interaction between the free-bacterial community and *L. polyedrum* still constitute a form of symbiosis between vitamin producing bacteria in suspension and *L. polyedrum* where the latter provides the labile organics to the media to sustain the growth of the suspended bacteria. We did not measure dissolved organic carbon (DOC) in the culture medium but the medium was prepared from aged seawater and no DOC was added by us. Our data do not exclude the possibility of vitamin acquisition by either episymbiosis or phagocytosis, but we found no microscopic evidence for phagocytosis. We considered episymbiosis to be unlikely because of similar numbers and phylogenetic composition of attached bacteria, but attached epibionts could still contribute to the B_12_ supply (Wagner-Döbler et al., 2010).

### Free and Attached Bacterial Community by FISH

The selected probes used for FISH analysis were based on available probes for bacteria associated with dinoflagellates. This method identified Alphaproteobacteria as the dominant bacterial group in the attached and freely suspended bacterial community in B-s and B-ns cultures (**Figures 4A,B**). It is difficult to relate the dominance observed in this study to particular functional phenotypes, because the Alphaproteobacteria are metabolically diverse (Luo and Moran, 2014). However, recent evidence indicates that the Alphaproteobacteria could contribute with B_1_ and B_12_ to their dinoflagellate host (Wagner-Döbler et al., 2010). The *Roseobacter* clade is an important marine Alphaproteobacteria lineage associated with dinoflagellates (Fandino et al., 2001; Hasegawa et al., 2007; Mayali et al., 2008, 2011) and includes species that produce B_1_ and B_12_ (Wagner-Döbler et al., 2010). Bacteria within this clade are known to be epibionts of dinoflagellates, including *L. polyedrum* (Mayali et al., 2011) and can represent the most abundant group within the bacterial assemblages associated with phytoplankton cultures and during bloom conditions (Fandino et al., 2001; Hasegawa et al., 2007).

We also identified Gammaproteobacteria and Bacteroidetes as less frequent epibionts. Gammaproteobacteria and Bacteroidetes have been found previously in natural samples during bloom conditions (Fandino et al., 2001; Garcés et al., 2007; Mayali et al., 2011), their low attachment frequency in our cultures is in agreement with their low abundances reported in culture and field samples (Garcés et al., 2007). Genomic data about these two groups confirm that most Gammaproteobacteria have the metabolic potential to produce B_1_ and B_12_, whereas only some Bacteroidetes possess the pathway for B_1_ but not for B_12_ (Sañudo-Wilhelmy et al., 2014).

The stable communities observed by FISH counts during the course of the culture (**Figures 4A,B**) suggest that Alphaproteobacteria and possibly *Roseobacter* clade species are an integral part of the epiphytic community of *L. polyedrum*. The similarity of probe frequency in attached and suspended fractions and in both vitamin amendments, suggests that bacteria of the different phylogenetic groups could move between both forms, thus, the bacterial community composition seemed more influenced by the host-specificity rather than the capacity to produce vitamins.

### Free and Attached Bacterial Community by 16S Amplicons

Overall, the phylogenetic composition of the four samples was restricted to the Alphaproteobacteria and Gammaproteobacteria classes and Bacteroidetes phylum. The Alphaproteobacteria class dominated in the free and attached bacterial fraction.

In this study, *Marivita* sp. was more abundant in the B-ns culture in comparison with the B-s culture, suggesting a functional association with *L. polyedrum* for example as B vitamin producer, or potentially as suggested in Green et al. (2015) as a growth-promoter. Wagner-Döbler et al. (2010) and Durham et al. (2015) observed other members of the *Roseobacter* clade to be associated with phytoplankton such as *Dinoroseobacter shibae* and *Ruegeria pomeroyi* respectively.

The second most abundant species in our samples was *Marinobacter flavimaris*. *Marinobacter* genus has been reported in association with dinoflagellates in culture (Green et al., 2004, 2006, 2015; Hatton et al., 2012). This genus is known for having a mutualistic relationship with phytoplankton as an iron siderophore producer or as a growth promoter (Amin et al., 2009; Bolch et al., 2011). During an iron-siderophore survey, *M*. *flavimaris* was isolated from different dinoflagellates species in culture but not from *L. polyedrum* (Amin et al., 2009). This species is phylogenetically related to *M. algicola, M. adhaerens*, and *M. aquaeolei* based on 16S rRNA sequence (Gärdes et al., 2010), however, its metabolic potential is expected to be rather different since *M*. *algicola* is known for siderophore production, *M*. *adhaerens* for the induction of marine organic matter aggregation and *M*. *aquaeolei* for its capacity to consume hydrocarbons. These different physiological profiles make it difficult to assume a metabolic potential for *M. flavimaris* based on the 16S rRNA gene.

In a recent study, Green et al. (2015) reported the co-occurrence of *Marivita* sp. and *Marinobacter* sp. in cultures of the coccolithophorids *Emiliania huxleyi* and *Coccolithus pelagicus* f. *braarudii*. In our data we have a similar co-occurrence of *Marivita* sp. and *Marinobacter flavimaris*; in the attached fraction, in the B-ns, the ratios were *Marivita* sp.: *M. flavimaris* 2.7:1, while in the B-s were 2.3:1. In the free-bacterial fraction, this ratio changed from 15.5:1 for the B-ns, to 1:1 for the B-s. Since iron was in replete conditions, our data suggest that this co-occurrence is more related to the vitamin supply.

The third most abundant species was from the phylum *Bacteroidetes*. This phylum has been recognized as specialist for the degradation of macromolecules, having a preference for growth attached to particles, surfaces or algal cells (Fernández-Gómez et al., 2013; Mann et al., 2013). Our data follow this pattern, since the attached fraction in B-s and B-ns cultures was enriched with member of this group. In the B-s condition, the *Bacteroidetes* accounted for 27%, mainly represented by *Fluviicola* sp., whereas in the B-ns culture, *Fluviicola* sp. represented 13.5% (**Figure 5**). This bacterial species has been previously reported during a *L. polyedrum* bloom, exhibiting its highest peak during the middle bloom stage, and a second peak at the end of the bloom, mainly clustered with other *Flavobacteria* species (Mayali et al., 2010, 2011).

**FIGURE 6:**
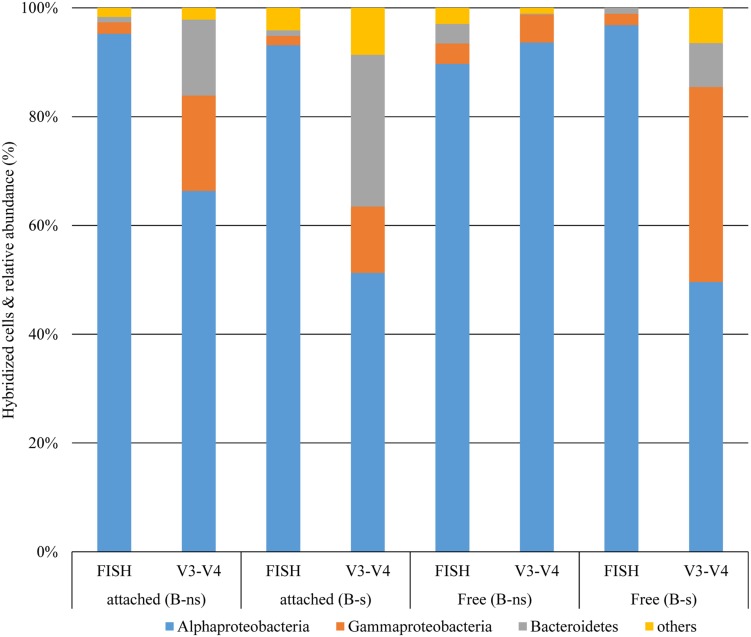
**Comparison of taxonomic classification of bacterial groups at phylum and class levels from 16 rDNA-FISH hybridization and 16S rDNA (V3–V4) Illumina reads**.

Other alphaproteobacterial species were detected in the samples. *Maricaulis* sp. has been detected associated with toxic and non-toxic dinoflagellates in culture (Hold et al., 2001; Strömpl et al., 2003) but its relation to dinoflagellate ecophysiology is not known. Another species was *Pelagibaca* sp., and despite its enrichment in the B-ns amendment, its closest relative, *Pelagibaca bermudensis* HTCC2601 has only been detected with a free-living lifestyle (Slightom and Buchan, 2009), although some members of the *Roseobacter* clade, are known to have the potential to synthesize cobalamin, thiamine and iron siderophores (Newton et al., 2010). *Erythrobacter* sp. was an infrequent species but showed >1% in the B-s attached fraction. *Erythrobacter* sp., a marine aerobic anoxygenic phototroph like *Dinoroseobacter shibae* (Koblížek et al., 2003; Wagner-Döbler et al., 2010), is known for its free-living lifestyle that is scarcely detected in dinoflagellate cultures (Green et al., 2015). Although FISH counts showed that the phylum Bacteroidetes was numerically less abundant in all dinoflagellate growth phases than other groups, the Illumina data showed that their contribution during exponential phase accounted for 13.98% of the attached bacteria (FISH 0.9%) for B-ns, and 27.8% (FISH 1%) in B-s; and for free-bacteria 0.1% (FISH 3.6%) for B-ns, and 8% (FISH 1%) for B-s (**Figure 6**).

The community composition obtained using FISH counts differed from the 16S rDNA amplicon reads (**Figure 6**). This type of difference has been observed before (Dawson et al., 2012; Lenchi et al., 2013). There are different possible explanations for these differences: FISH probe hybridization efficiency or the relative amount of inactive cells that could not be visualized by FISH were probably not responsible for the difference because the FISH count were checked against the eubacterial probe EUB338 and DAPI staining showing that most prokaryotes were stained by FISH (**Figures 4A,B**). Another explanation for the difference might have been the low coverage of the probe for the represented groups in the culture, given that probe ALF968 covers approximately 81% of the class Alphaproteobacteria, probe GAM42a covers approximately 76% of the class Gammaproteobacteria, and CF319a covers approximately 38% of the phylum Bacteroidetes (Amann and Fuchs, 2008). Because we were working with probes previously used in research related to the bacterial diversity associated with dinoflagellates (Alavi et al., 2001; Alverca et al., 2002; Biegala et al., 2002; Garcés et al., 2007; Cruz-López and Maske, 2014) we did not confirm the selected probes targeted sequences in our Illumina data set before FISH experiments. One simple reason for the difference could be that the primers used to target the V3-V4 region (V3 338-533, V4 576-682; *Escherichia coli* 16S SSU rDNA numbering) and the FISH probes covered different regions or even the 23S rDNA subunit (**Table 1**) did not allow us to make a direct correlation between the two techniques. Dawson et al. (2012) and Lenchi et al. (2013), suggested different explanations for the difference, the underrepresentation of some phyla, PCR primer or gene copy number bias in the 16S rRNA sequencing, or the possibility of bacterial groups not being properly covered by the probe combination used, and the low relative abundance arguments that we would reject for reasons mentioned above.

### Dissolved B_12_ Synthesis from the Microbial Community

Our results show that although the concentration of B_12_ in the B-s culture was higher than in the B-ns culture, this difference should be the result of the initial supplemented concentration from L1. The B-s culture medium contained initially 370 pmol L^–1^ of B_12_ that was reduced to 26 pmol L^–1^ during culture growth, close to twice the concentration in the exponential phase B-ns culture of 14.4 pmol L^–1^. The high consumption of B_12_ in the B-s culture might be due not only to *L. polyedrum* but also to the bacterial community. Neither of the culture concentrations was expected to be limiting the growth rate of *L. polyedrum* because these concentrations were as high as in very productive coastal waters. In the case of the B-ns culture the source of B_12_ was the microbial community maintaining the exponential growth phase of *L. polyedrum* while being supported by organic substrate provided by the phototroph host. The *L. polyedrum* biomass in B-ns and B-s cultures during vitamin sampling was similar. If we assume that the vitamin consumption is proportional to *L. polyedrum* biomass, neglecting B_12_ luxury uptake by *L. polyedrum*, consumption by bacteria or the production by bacteria in the B-s culture. From this we can roughly estimate that 370 –26 pmol L^–1^ of B_12_ was produced by the bacterial community during B-ns culture development to support the growth 8 × 10^6^ *L. polyedrum* cells L^–1^ (**Supplementary Figure S1**), which amounts to 4 × 10^–17^ mol B_12_ cell^–1^. This high potential of B_12_ production while being supported by organics provided by the auxotrophic *L. polyedrum* suggests an easy capacity for the bacterial community to supply the oceanic auxotrophs phototrophs with B_12_ as long as no other controls limit the bacterial development. Our data suggest that B_12_ synthesis in the *L. polyedrum*/bacterial community respond to changes in B_12_ concentration, and that the supply may depend on the extent of B_1_ and B_12_ limitation, which in turn selects the bacterial community structure and it B_1_ and B_12_ synthesis capacity. The next step to study the contribution of all players within this type of system would be the use of metatranscriptomics combined with mass-spectrometry for the quantification of gene expression levels and its products, to estimate the contribution of eukaryotic DOC exudation and bacterial products.

## Conclusion

In this work we have shown that *L. polyedrum* is a B_1_ and B_12_ auxotroph. Non-axenic cultures of *L. polyedrum* can acquire both vitamins from the associated bacterial community in sufficient quantity to sustain the maximum growth rate defined by culture conditions. The growth of the associated heterotrophic bacterial community was sustained by substrates provided by *L. polyedrum* because the culture medium was not amended with organic substrates.

Previous studies have shown B_12_ auxotrophy of dinoflagellates and diatoms species and the acquisition of B_12_ from a bacterial partner, however, is its known that, phytoplankton such as dinoflagellates not only require B_12_, but also B_1_, and they naturally interact with bacterial and archaeal communities, rather than with single bacterial species.

A small portion of the associated bacterial community was attached to the *L. polyedrum* cells; with or without vitamin addition to the culture medium the statistics of attachment, the proportion of *L. polyedrum* cells with bacteria attached or the number of bacteria attached to *L. polyedrum* cells did not change, but the abundance of freely suspended bacteria was significantly higher in B-ns culture. Using the same bacterial community inoculum, we showed that a characteristic bacterial community emerges even under contrasting vitamin amendments. We demonstrate that by association, the microbial community in the B-ns culture was able to synthesize and excrete sufficient B_1_ and B_12_ quantities to sustain the growth of its dinoflagellate host while being supported by organic substrates supplied by the host.

## Author Contributions

All authors listed, have made substantial, direct and intellectual contribution to the work, and approved it for publication.

## Conflict of Interest Statement

The authors declare that the research was conducted in the absence of any commercial or financial relationships that could be construed as a potential conflict of interest.

## Supplementary Material

FIGURE S1 **Growth of *L. polyedrum* and associated bacteria culture under B-s () and B-ns () conditions. (A)** *L. polyedrum* abundance. **(B)** Free-bacteria abundance. **(C)** Percent of *L. polyedrum* cells colonized by bacteria.
